# Unlocking the bovine genome

**DOI:** 10.1186/1471-2164-10-193

**Published:** 2009-04-24

**Authors:** Ross L Tellam, Danielle G Lemay, Curtis P Van Tassell, Harris A Lewin, Kim C Worley, Christine G Elsik

**Affiliations:** 1 CSIRO Livestock Industries, 306 Carmody Rd, St Lucia 4067, QLD, Australia; 2 Department of Food Science and Technology, University of California, One Shields Ave, Davis, CA 95616, USA; 3 Bovine Functional Genomics laboratory, USDA/ARS, BARC-East, Beltsville, MD, 20705, USA; 4 Institute for Genomic Biology and Department of Animal Sciences, University of Illinois at Urbana-Champaign, Urbana, IL 61801, USA; 5 Human Genome Sequencing Center, Department of Molecular and Human Genetics, Baylor College of Medicine, One Baylor Plaza, MS BCM226, Houston, TX 77030, USA; 6 Department of Biology, 406 Reiss, 37th & O Streets NW, Georgetown University, Washington, DC 20057, USA

## Abstract

The draft genome sequence of cattle (*Bos taurus*) has now been analyzed by the Bovine Genome Sequencing and Analysis Consortium and the Bovine HapMap Consortium, which together represent an extensive collaboration involving more than 300 scientists from 25 different countries.

## Commentary

The cattle genome [1,2] was originally selected for sequencing because of the unique biology of ruminants, the importance of ruminants as a major source of protein nutrition for humans, and the evolutionary position of cattle relative to other mammals [3]. Wide-ranging and detailed analyses of the genome sequence and population based variation within the sequence are reported in 22 companion publications, the majority of which appear in the *BioMed Central *group of journals published in April 2009, appropriately, the Year of the Ox. A full list of companion papers and other materials can be found at [4]. These reports provide remarkable insight into mammalian evolution, ruminant biology, and the origins of cattle breeds and impacts of domestication on the species.

At present, there are approximately 3.4 billion domesticated cattle, buffalo, sheep and goats in the world which provide a major source of protein nutrition for 6.6 billion humans [5]. About three quarters of the world's agricultural land produces forage suitable for grazing by these ruminants, which have an astonishing capacity to efficiently convert low quality plant fibre that is often unsuitable for human use into energy dense fat, muscle and milk. This biological process has long been exploited by humans. Ruminants are also valued for their hides, hair and work capacity. Indeed, the social and cultural evolution of humans, particularly as they adopted an agrarian lifestyle, is intimately intertwined with ruminant domestication, which began in the Near East some 8,000–10,000 years ago [6,7]. The bull and cow have been symbols of human prosperity and political power and, in some cultures, have attained the status of religious icons.

The growth of domesticated ruminant populations, especially cattle, has mirrored the rapid expansion of the human population. More than 800 cattle breeds have been selected by humans since domestication for different economic, social and religious reasons and these represent an important world heritage and a unique scientific resource [8]. While the hand of humans has certainly sculpted the genetic architectures of modern cattle breeds, it is interesting to note that the human genome has also undergone selective adaptation for tolerance to lactose in response to domestication of ruminants for milk production [9].

The cattle genome sequence fills an evolutionary void among mammalian genome sequences as it is the first complete high coverage genome sequence from the Cetartiodactyl order of eutherian mammals which first appeared ~60 million years ago when grasslands began to cover large parts of the earth's surface. The order includes ~220 species of even-toed ungulate mammals (suborders Ruminantia, Suina and Tylopoda), hippopotamus and 88 cetacean species (whales, dolphins and porpoises). The Cetartiodactyla have adapted to an extraordinary diversity of terrestrial and aquatic environments. Cattle are representative of the Ruminantia, a phylogenetically distant clade to humans and rodents, the two most intensively investigated mammalian species. The evolutionary relationships between these three mammalian groups are well positioned to inform genome similarities and differences in each group and comment on mammalian biology. The evolution of genome organization, particularly the expansion and contraction of paralogous gene families and the molecular bases for economically important adaptive traits of ruminants such as lactation, metabolism, reproduction and disease resistance, are all important scientific issues that can explicitly be addressed by comparative analysis of the cattle genome with other mammalian genomes.

The unique biology of ruminants was a predominant factor influencing the decision to sequence the cattle genome [3]. Ruminants are characterized by their ability to convert ingested complex plant carbohydrates into volatile fatty acids such as acetate, propionate, butyrate, lactate and valerate, which are then used as the major energy source [10]. This remarkable fermentative transformation is performed by a complex community of symbiotic microorganisms present in the rumen. Microbial fermentation of plant material is also the main source of dietary protein and all B vitamins [10]. The immune system of cattle appears to be exquisitely adapted to the extensive microbial communities that populate its gut and exposed epithelial surfaces. For example, up to 40% of peripheral blood lymphocytes of cattle have γ/δ T-cell receptors as compared to less than 1% in humans and rodents [11]. Thus, cattle are an excellent model species to study the evolution and function of these enigmatic γ/δ T-cells, which appear to play a major role in immune regulation and suppression. Cattle are also used as models for human reproductive biology and infectious diseases. The latter application is also critical to maintaining a safe, secure and healthy food supply. Assisted reproductive technologies used by humans such as artificial insemination, estrous synchronization, embryo transfer and in vitro fertilization, were first developed for use in cattle. Furthermore, cattle and other ruminants can be efficiently cloned from adult somatic cells and therefore they represent excellent models to decipher the roles of epigenetic modifications on development and differentiation of stem cells [12].

The assembled 7.1× cattle genome sequence primarily represents DNA from a single inbred female Hereford animal, L1 Dominette [1,13,14]. In addition, limited sequence information was obtained from individuals representing six breeds, which allowed identification of putative SNP, a major resource for the HapMap Project which used 34,470 of these in its analyses. The sequencing, assembly, placement of sequence scaffolds on bovine chromosomes and assessment of assembly quality were assisted by a range of livestock research community resources including radiation hybrid maps, chromosome specific and whole genome linkage maps, physical maps based on BAC end sequence information, extensive EST collections and approximately 10,000 full length cDNA sequences [1,13]. An independent high density linkage map of the bovine genome is also reported [15]. The Bovine Genome Sequencing and Analysis Consortium and the Bovine HapMap Consortium used a variety of genome wide computational approaches, comparative genomics analyses and focused gene specific investigations to highlight the evolutionary history, unique biology and population diversity associated with modern cattle.

What has the initial analysis of the bovine genome sequence revealed? Evolutionary breakpoint regions in mammalian chromosomes, which define the boundaries of conserved syntenic blocks of sequence, are associated with considerable evolutionary plasticity in mammals. Large segmental duplications are over-represented and some types of repetitive sequence elements are selectively enriched or excluded in these regions [1,16]. Moreover, the segmental duplications are enriched for genes whose protein products often directly interface with the external environment e.g. immune proteins and sensory receptors. This observation suggests that these genes represent evolutionary adaptations to the immediate environment of cattle. Comparative analyses also revealed that sequences of bovine proteins are generally more similar to human orthologs than are rodent orthologs. There are also cattle specific changes in the organization of genes involved in digestion, immunity, reproduction and lactation [1].

Milk is exquisitely formulated to suit the differing developmental needs of newborn mammals. Analysis of the genes encoding proteins present in bovine milk and the genes expressed in mammary tissue during the lactation cycle provide the first comprehensive overview of lactation genomics [17]. The integration of this information with population genetic resources generated in the dairy industry provides an enabling resource for future discovery of genes underpinning complex dairy production traits. These analyses clearly indicate that milk is not just a convenient source of high quality nutrients but it also contains a variety of bioactive proteins which probably play key roles in the antimicrobial defense system of the newborn suckling calf and possibly also in gut maturation.

Several of the companion papers focused on changes in the organization of specific gene families in the bovine lineage and their potential impact on ruminant biology. Compared with humans and rodents, there are significant changes in the organization of several immune gene families [1] exemplified by the serum amyloid A3 [18] and type 1 interferon [19] families, as well as the major histocompatability complex [20] and T cell receptor B locus [21]. The extensive genomic rearrangements of these and other immune gene families suggest that during evolution of the bovine lineage there has been adaptation to changing infectious disease challenges [1]. There are also alterations in the organization of gene families involved in reproduction compared with the human and rodent genomes [22]. These probably reflect the distinctive reproductive program in cattle, particularly differences in placental structure. In addition, a number of genes are implicated in various aspects of bovine reproductive function and early development [23,24].

Metabolic reconstruction analysis revealed that most genes encoding metabolic enzymes are conserved in mammals however in cattle a few gene losses compared with other mammalian species were identified [25]. These evolutionary changes may be important for understanding metabolic adaptations to the ruminant lifestyle. Examination of defined regions of chromosome 6 revealed a number of novel transcripts [26] suggesting that the full complement of transcriptional activity of the genome is yet to be defined. This is a reminder of a major future challenge – the mapping of the complete repertoire of transcriptional activity across the genome.

The Bovine HapMap Consortium analysed the frequency of over 37,000 SNP in 497 cattle from 19 geographically and biologically diverse cattle breeds [2]. The resulting hapmap is literally a map of the genetic diversity among different cattle populations. Analysis of the hapmap revealed a picture of cattle history in which the ancient population size was relatively large but genetic diversity was dramatically reduced by the processes of domestication and selective breeding [2]. Thus, while the overall number of domesticated cattle has increased in parallel with the expanding human population, the genetic diversity of those animals has decreased. Nevertheless, genetic diversity among cattle remains greater than among dog breeds and is similar to the diversity among humans [2]. In a companion report, the haplotype structures for several cattle breeds were determined at high resolution [27]. Other reports describe the use of population genetics to investigate the origins and evolution of the different breeds [28,29], effective population sizes [30] and signatures of positive selection associated with domestication [31]. Another report highlights the potential for direct industry application of population genetic information, namely the association of variation of the Integrin Beta 5 gene with bull fertility [32]. A cattle QTL meta-analysis was also performed to identify QTL confirmed in multiple studies using different populations [33]. This analysis provides the scientific community with additional confidence to pursue the identification of causal genetic polymorphisms underlying many complex production traits. Another report describes the development and performance of a higher-density SNP assay that leveraged the information gleaned from the HapMap Consortium efforts [34].

Although these collective studies generated a multitude of discoveries, this is only the beginning of a much larger body of future research that will be catalyzed by the availability of the bovine genome sequence and haplotype map. The genome sequence will be refined, reanalyzed and integrated with biological data in ways we can not yet imagine to examine fundamental questions relating to the linkage between genes and cattle phenotypes, the reasons for the evolutionary success of ruminants, the history of cattle domestication, and the mechanisms of mammalian evolution, to name but a few. The availability of very large well managed cattle populations and sire based breeding systems will accelerate the discovery of genes contributing to complex traits of direct relevance to humans such as energy partitioning, muscle formation, milk production and disease resistance. Undoubtedly, the bovine genome sequence and haplotype map will revolutionize the beef and dairy industries by providing genetic and genomic tools to help address the pivotal livestock issues of the 21^st ^century – efficient and sustainable production systems with smaller environmental footprints.
